# Accuracy of the Electronic Nose Breath Tests in Clinical Application: A Systematic Review and Meta-Analysis

**DOI:** 10.3390/bios11110469

**Published:** 2021-11-22

**Authors:** Hsiao-Yu Yang, Wan-Chin Chen, Rodger-Chen Tsai

**Affiliations:** 1 Institute of Environmental and Occupational Health Sciences, National Taiwan University, Taipei 10055, Taiwan; 117152@cch.org.tw (W.-C.C.); ricxeor@gmail.com (R.-C.T.); 2 Department of Public Health, National Taiwan University College of Public Health, Taipei 10055, Taiwan; 3 Department of Environmental and Occupational Medicine, National Taiwan University Hospital, Taipei 10002, Taiwan; 4 Department of Family Medicine, Changhua Christian Hospital, Changhua 50006, Taiwan

**Keywords:** volatile organic compound, electronic nose, sensors, breath test, breathomics

## Abstract

(1) Background: An electronic nose applies a sensor array to detect volatile biomarkers in exhaled breath to diagnose diseases. The overall diagnostic accuracy remains unknown. The objective of this review was to provide an estimate of the diagnostic accuracy of sensor-based breath tests for the diagnosis of diseases. (2) Methods: We searched the PubMed and Web of Science databases for studies published between 1 January 2010 and 14 October 2021. The search was limited to human studies published in the English language. Clinical trials were not included in this review. (3) Results: Of the 2418 records identified, 44 publications were eligible, and 5728 patients were included in the final analyses. The pooled sensitivity was 90.0% (95% CI, 86.3–92.8%, I^2^ = 47.7%), the specificity was 88.4% (95% CI, 87.1–89.5%, I^2^ = 81.4%), and the pooled area under the curve was 0.93 (95% CI 0.91–0.95). (4) Conclusion: The findings of our review suggest that a standardized report of diagnostic accuracy and a report of the accuracy in a test set are needed. Sensor array systems of electronic noses have the potential for noninvasiveness at the point-of-care in hospitals. Nevertheless, the procedure for reporting the accuracy of a diagnostic test must be standardized.

## 1. Introduction

Human exhaled breath contains numerous volatile metabolites produced during diseases’ physiological and pathological processes that can be used as volatile biomarkers for diagnosis [1]. Breathomics is an emerging science to diagnose diseases by analyzing volatile organic compounds (VOCs) produced by changes in metabolic processes caused by disease [1]. The electronic nose (E-nose) and gas chromatography-mass spectrometry (GC-MS) are two methods to analyze these VOCs. In contrast to the E-nose, GC-MS allows us to explore possible biological pathways and identify specific VOCs associated with the pathological changes of the diseases. The E-nose aims to develop point-of-care diagnostic breath tests [2]. The E-nose uses a nonselective sensor array to identify the pattern of VOCs in exhaled breath. When VOCs from a breath sample is presented to the sensor array, the E-nose system processes the response signals of sensor arrays and uses machine learning techniques to discriminate the VOCs of patients from healthy subjects and provides the most likely diagnosis based on smell [3]. Currently, the E-nose has been implemented in the diagnosis of lung cancer [4], breast cancer [5], colorectal cancer [6], ovarian cancer [7], gastric cancer [8], head-and-neck cancer [9], chronic obstructive lung disease (COPD) [10], interstitial lung disease [11], liver cirrhosis [12], ventilator-associated pneumonia [13], and Coronavirus Disease 2019 (COVID-19) [14]. In artificial intelligence (AI), the development of electronic nose systems is an emerging science that can provide real-time analysis and assist clinical decisions. There are two major types of sensors: (1) nanomaterial-based sensors, including single-walled carbon nanotubes (CNTs), monolayer capped metal nanoparticle (MCNP) films and metal oxide (MO) sensors, and (2) electroacoustic sensors that include quartz microbalance (QMB) and surface acoustic wave (SAW) sensors [15].

The current knowledge gap on the application of E-noses to clinical diagnosis remains uncertain. Due to the advancement of material sciences, many types of E-nose sensors have been developed in recent years [16]. Although many types of sensors have been designed to detect more diseases in recent years, E-noses have not yet been applied in clinical practice. An updated systemic review and meta-analysis are necessary to provide quantitative and qualitative estimates of the accuracy of the E-nose in actual patients.

The specific aims of this review were to (1) summarize the diagnostic accuracy of sensor-based exhaled breath tests for clinical diagnoses and (2) compare the accuracy of different types of sensors. To achieve these aims, we performed a systematic review of the published evidence regarding the use of the sensors in breath tests for clinical diagnosis.

## 2. Materials and Methods

This meta-analysis was conducted following the PRISMA 2020 guidelines for reporting systematic reviews [17]. The review included only studies that analyzed VOCs in the exhaled breath of human subjects in hospitals. Studies that involved cell lines or animal studies were excluded. All analyses were based on previously published studies, and thus, no ethical approval or patient consent was required.

### 2.1. Eligibility Criteria

Studies were included if they met the following criteria: (1) the study analyzed VOCs within exhaled breath; (2) the study was an observational study, with a cross-sectional, case-control, or prospective design; and (3) the study’s population consisted of patients or healthy controls enrolled from hospitals. The exclusion criteria were as follows: (1) in vitro experiments; (2) animal studies; (3) studies in which VOCs were analyzed not in exhaled breath but in breath condensate or tissue, including urine, blood, stool, or other biofluids; (4) reports not published in the English language; (5) studies of laboratory testing of sensor prototypes that were not applied in a clinical setting; (6) duplicate publications; (7) letters or review articles; and (8) studies that did not provide sufficient information on case number, control number, sensitivity, and specificity to construct the 2 × 2 contingency table.

### 2.2. Information Sources

We selected related studies published between 1 January 2000 and 14 October 2021 by searching PubMed and Web of Science. We also searched documents that cited any of the initially included studies as well as the references of the initially included studies.

### 2.3. Search Strategy

**Figure 1:**
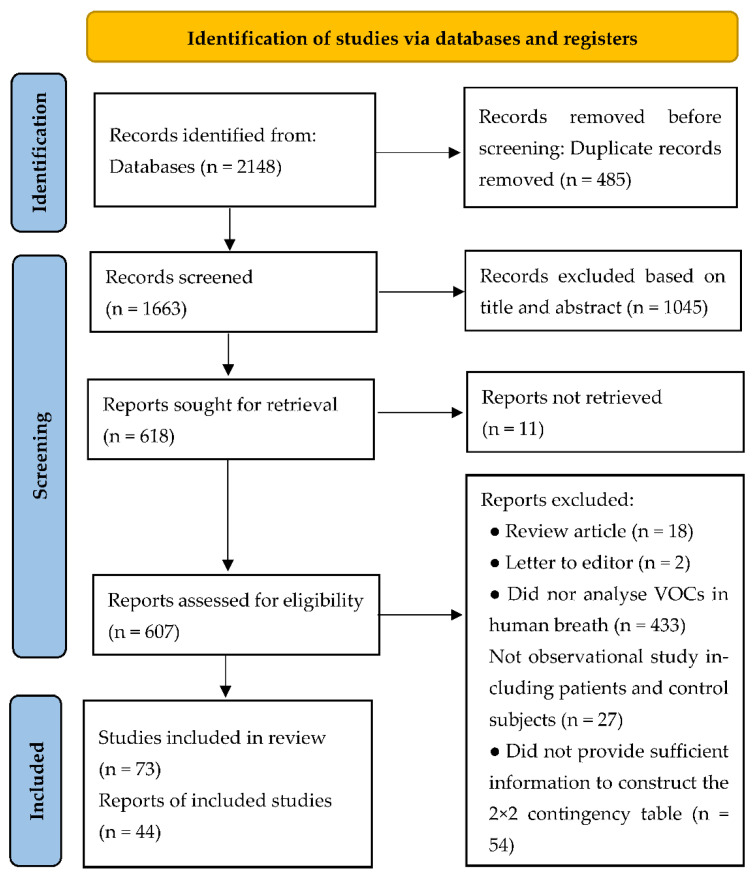
PRISMA flow chart of literature search.

We used the following combined text in Web of Science: (ALL = (breath analysis OR breath test)) AND ALL = (sensor). The complete search used for PubMed was: Search: (breath analysis OR breath test) AND (sensor) Filters: from 2010–2022 ((“breath tests”[MeSH Terms] OR (“breath”[All Fields] AND “tests”[All Fields]) OR “breath tests”[All Fields] OR (“breath”[All Fields] AND “analysis”[All Fields]) OR “breath analysis”[All Fields] OR (“breath tests”[MeSH Terms] OR (“breath”[All Fields] AND “tests”[All Fields]) OR “breath tests”[All Fields] OR (“breath”[All Fields] AND “test”[All Fields]) OR “breath test”[All Fields])) AND (“sensor”[All Fields] OR “sensors”[All Fields] OR “sensoric”[All Fields] OR “sensorics”[All Fields] OR “sensoring”[All Fields] OR “sensorization”[All Fields] OR “sensorized”[All Fields] OR “sensors”[All Fields])) AND (2010:2022[pdat]). Furthermore, the reference lists of relevant articles were manually examined to determine additional potentially related studies. The searches were carried out independently by two investigators (H.-Y.Y. and R.C.T.). Later, we searched documents that cited any of the initially included studies as well as the references of the initially included studies. However, no extra articles that fulfilled the inclusion criteria were found in these searches. Full details of the search strategy are provided in Figure 1.

### 2.4. Selection Process

Three investigators (R.-C.T., W.-C.C.) independently reviewed the study titles and abstracts at first and discussed the inconsistencies until consensus was obtained. Disagreements were resolved by consensus and discussion with the corresponding author (H.-Y.Y.). We contacted the corresponding author if further information was needed. If no response was received, the study was excluded from the meta-analysis.

### 2.5. Data Items

We extracted the following study characteristics from each eligible study: the name of the first author, publication year, country, disease, number of participants, and type of sensor. Each investigator also recorded or calculated the number of false positives (FPs), true positives (TPs), false negatives (FNs), and true negatives (TNs). For studies that reported the results of different machine learning algorithms, we selected the best results for the meta-analysis. For studies with multiple comparison groups (i.e., cancer, benign disease, and healthy controls), we derived data from the primary disease and healthy controls.

### 2.6. Quality Assessment

We used a modified Quality Assessment of Diagnostic Accuracy Studies 2 (QUADAS-2) tool to assess the quality of the included studies. QUADAS-2 consists of four domains, including patient selection, index test, reference standard, and flow of patients through the study [18]. Two reviewers (H.-Y.Y. and W.-C.C.) independently rated all included studies. We used the QUADAS-2 sheet built in the RevMan 5.3 software of Cochrane to provide a methodological quality summary [19].

### 2.7. Statistical Analysis

We obtained the numbers of FPs, TPs, FNs, and TNs to calculate the pooled point estimates of sensitivity, specificity, and summary ROC curve of breath tests [20]. ROC values of 0.7–0.8, 0.8–0.9, and 0.9–1 are regarded as good, very good, and excellent diagnostic accuracy, respectively [21]. Statistical heterogeneity caused by non-threshold effects was tested by the Q test and I^2^ test. An I^2^ value greater than 50% was considered to indicate significant heterogeneity [22]. If considerable heterogeneity could not be eliminated, a random-effects model was used [23]. We generated funnel plots to evaluate small study effects and applied Egger’s test to assess funnel plot asymmetry [24]. Because simple pooling of sensitivity and specificity is usually inappropriate, as this approach ignores threshold differences, we also calculated the diagnostic odds ratio (DOR):

(1) $$\mathrm{DOR}=\frac{\mathrm{TP}}{\mathrm{FP}}/\frac{\mathrm{FN}}{\mathrm{TN}}$$

The DOR is not prevalence dependent and offers considerable advantages in a meta-analysis of diagnostic studies with increased precision. The value of a DOR ranges from 0 to infinity, with higher values indicating better discriminatory test performance [25]. Because the accuracy in the test set is usually lower than that in the overall dataset, we compared the accuracy from the overall dataset and test set.

### 2.8. Sensitivity Analysis

We conducted sensitivity analyses to determine if there was an undue influence of publication bias on the pooled estimates of accuracy. We deleted studies when the number of FNs or FPs was less than or equal to zero or one, which resulted in a DOR higher than 300. We also restricted the studies to low risk of bias, in which there was less than or equal to one high-risk QUADAS-2 domain, to see the influence of bias from patient selection, index test, reference standard, and flow of patients through the study.

### 2.9. Subgroup Analysis

Subgroup analysis was performed to explore the sources of heterogeneity according to the characteristics of the included articles. We conducted a subgroup analysis to compare the DOR of different types of sensors.

We performed the meta-analysis with the R packages of MADA, META, and Metafore and Review Manager 5 software. A two-tailed *p*-value less than 0.05 was considered statistically significant.

## 3. Results

The review identified 44 relevant publications and 5728 subjects (Figure 1). The number of subjects included in those studies ranged from 25 to 1167 (median 82). Within included studies, aneurysm (n = 1), appendicitis (n = 1), asthma (n = 1), breast cancer (n = 2), bronchial and laryngeal cancer (n = 1), colorectal cancer (n = 1), chronic obstructive pulmonary disease (COPD) (n = 1), COPD and lung cancer (n = 1), cutaneous leishmaniasis (n = 1), echinococcosis (n = 1), epilepsy (n = 1), gastric cancer (n = 3), hemodialysis (n = 1), head and neck cancer (n = 2), head-and-neck cancer and lung cancer (n = 1), heart failure (n = 1), interstitial lung disease (n = 1), liver cirrhosis (n = 2), lung cancer (n = 7), multiple sclerosis (n = 2), ovarian cancer (n = 2), Parkinson’s disease (n = 1), pneumoconiosis (n = 1), preeclampsia (n = 1), rhinosinusitis (n = 1), COVID-19 (n = 3), small airway dysfunction (n = 1), and ventilator-associated pneumonia (n = 2) were studied. The most common type of sensor was metal oxide (n = 11), followed by carbon nanotubes (n = 9), gold nanoparticles and carbon nanotubes (n = 7), gold nanoparticles (n = 6), quartz microbalances (n = 5), metal nanoparticles (n = 2), organic polymers (n = 1), polycyclic aromatic hydrocarbons and single-wall carbon nanotubes (n = 1), and WO3 nanowires (n = 1) (Table 1).

### 3.1. Pooled Sensitivity, Specificity, ROC and DOR

**Figure 2:**
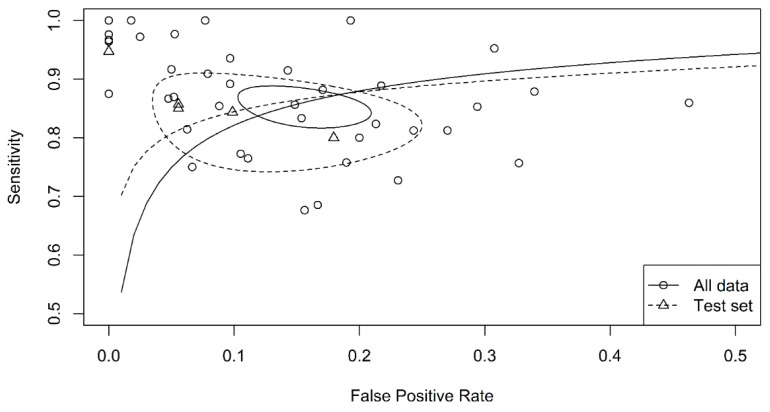
Summary receiver operating characteristic curve graph of the included studies. The accuracy using all data was higher than that of the test set.

**Figure 3:**
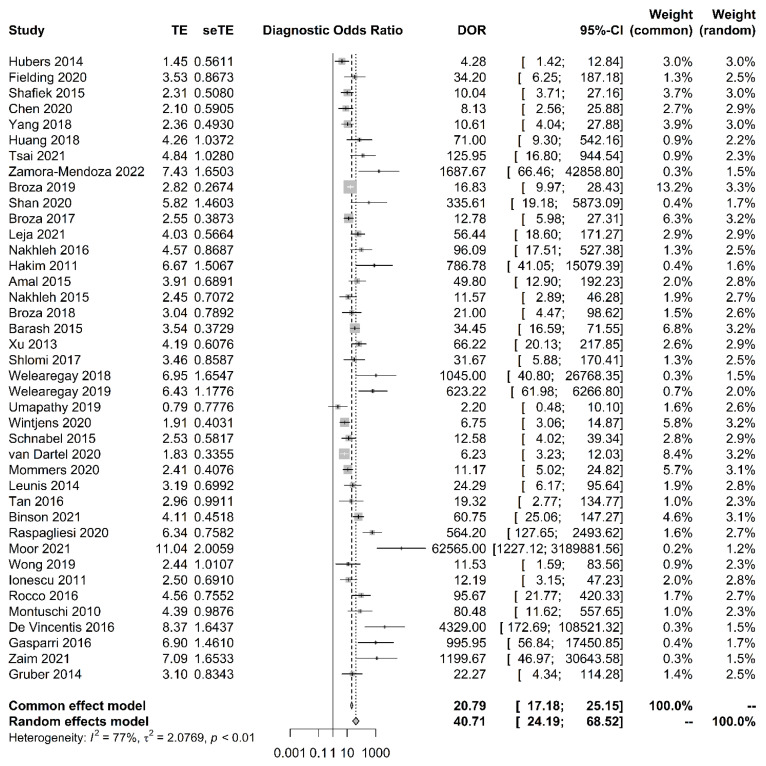
Forest plot and pooled diagnostic odds ratio analysis. Vertical dashed lines indicate 95% CI for the pooled effect. The size of the data markers reflects the weight. Error bars indicate 95% CI.

**Figure 4:**
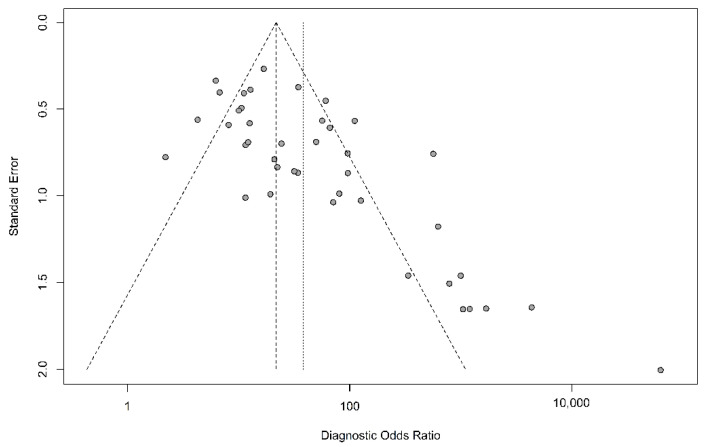
Funnel plot of the diagnostic odds ratio. A skewed asymmetrical funnel plot shows that there is publication bias. In the right lower corner, the small sample size studies (therefore large standard error) are more prone to publication bias than large studies.

The sensitivity of breath tests by sensor arrays ranged from 67.6% to 100%, whereas the specificity ranged from 29.4% to 100%. The pooled sensitivity was 90.0% (95% CI, 86.3–92.8%, I^2^ = 47.7%), the specificity was 88.4% (95% CI, 87.1–89.5%, I^2^ = 81.4%), the pooled area under the curve of 0.93 (95% CI 0.91–0.95) (Figure 2), and the pooled DOR was 40.7 (95% CI 24.2–68.5, I^2^ = 77.0%) (Figure 3). The funnel plot asymmetry and linear regression test (*p* value < 0.05) suggested potential publication bias (Figure 4).

### 3.2. Quality Assessment

**Figure 5:**
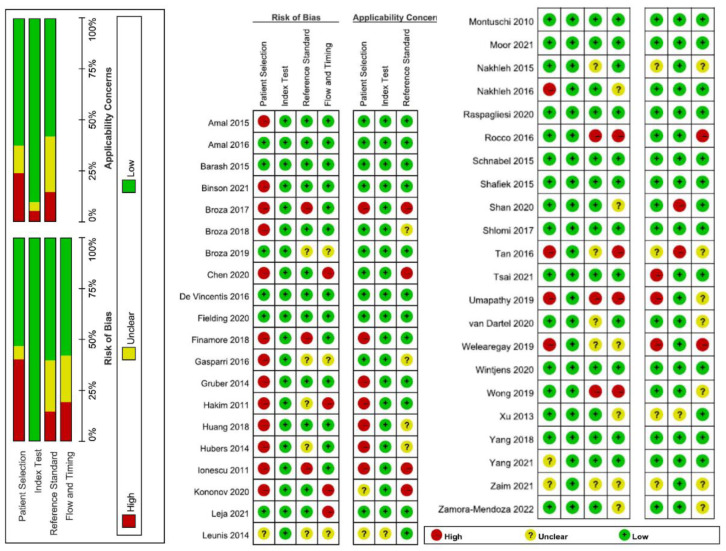
Quality assessment of included studies by the QUADAS-2 tool. This figure shows the proportion of studies with low (green colour), unclear (yellow), and high risk/concern (red). In terms of the overall risk of bias, there were concerns about the risk of bias for 26.5% of the studies (13/44), with two of these assessed as at high risk of bias.

The assessment of biases and applicability to outcomes utilizing QUADAS-2 are detailed in Figure 5. Major sources of bias were patient selection, followed by failing to report the reference standard, and flow and timing. The patient selection also became the major applicability concern for the E-nose test.

### 3.3. Sensitivity Analysis

After excluding 14 studies that reported that the number of false negatives or false positives was less than or equal to zero or one, the funnel plot was symmetric without publication bias (linear regression test of funnel plot asymmetry, *p* = 0.07), suggesting that the influence of these studies on the pooled results was acceptable, and the pooled results were robust to some extent. The pooled sensitivity was 83.4% (95% CI 80.3–86.1%, I^2^ = 38.7%), pooled specificity was 82.3% (95% CI 76.8–86.7%, I^2^ = 83.4%), pooled DOR was 19.3 (95% CI 13.6–27.4, I^2^ = 61.7%), and pooled area under the curve was 0.91 (95% CI 0.89–0.93). When restricting the analysis to studies considered to be at low risk of bias (n = 27), the pooled sensitivity was 90.8% (95% CI 86.0–94.0%, I^2^ = 56.5%), pooled specificity was 89.4% (95% CI 87.9–90.8%, I^2^ = 81.4%), and pooled area under the curve was 0.93 (95% CI 0.91–0.96).

### 3.4. Subgroup Analysis

**Figure 6:**
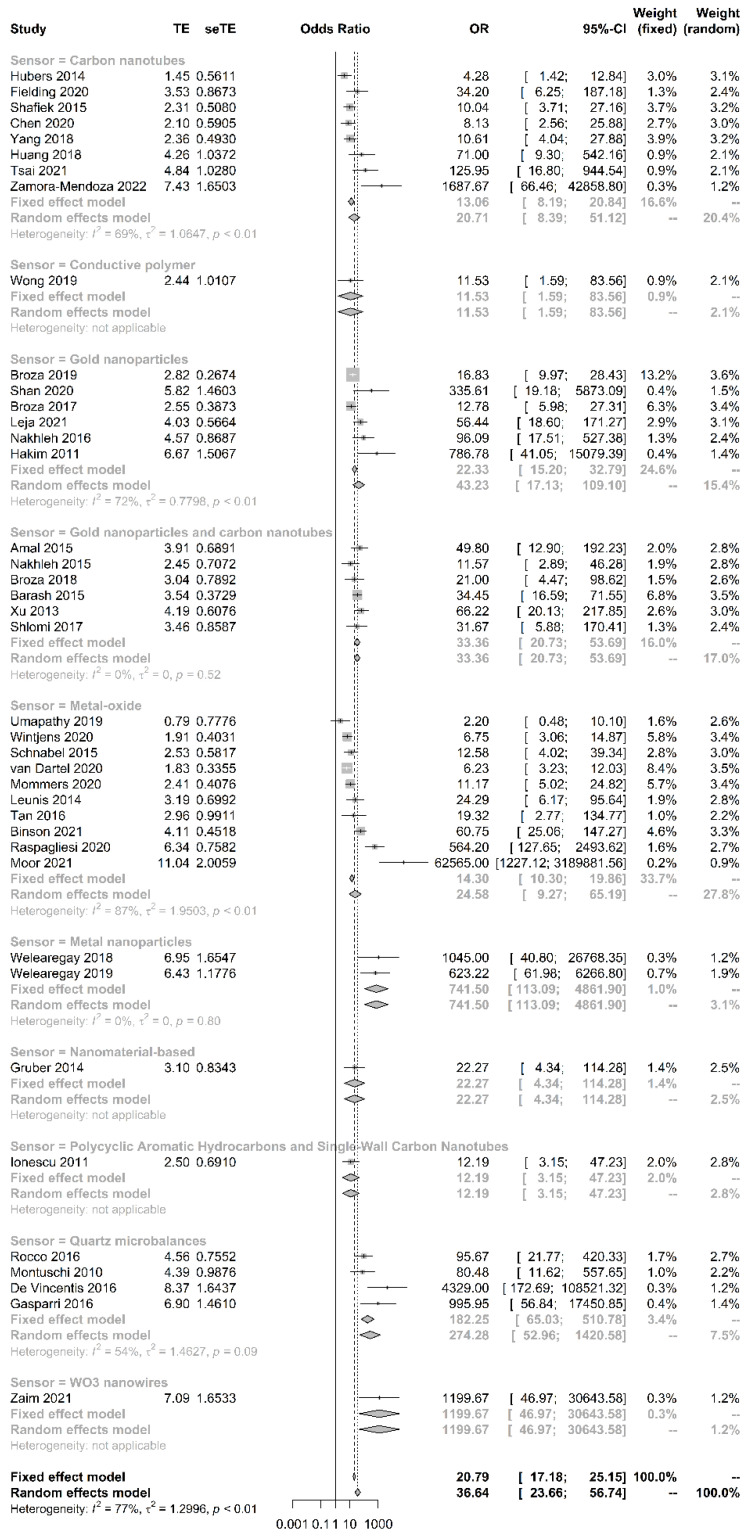
Subgroup analysis for pooled diagnostic odds ratio based on the type of sensor. The type of sensor is based on the classification provided in the literature.

We compared the accuracy of different types of sensors. Metal nanoparticle sensors had the highest DOR, sensitivity, and specificity (Table 2) (Figure 6).

## 4. Discussion

### 4.1. Summary of Main Results

This study provided evidence that the electronic nose analysis of exhaled breath has high accuracy in detecting diseases in actual patients. To the best of our limited knowledge, this is the first study to provide an overall estimate of the accuracy of the E-nose in clinical practice.

### 4.2. Strengths of the Review

This is the first study to provide a comprehensive review and pooled estimates of the diagnostic accuracy of E-noses in a clinical setting. This review provides quantitative estimates of the accuracy of different sensors, which will provide a basis for future researchers to choose suitable sensors and estimate the required sample size. There was moderate heterogeneity in the pooled sensitivity (I^2^ = 47.7%, *p* < 0.05) and high heterogeneity in specificity (I^2^ = 81.4%, *p* < 0.05). After we ruled out studies that reported extraordinarily high-accuracy studies to obtain a more reliable accuracy in clinical breath tests, the heterogeneity was improved and showed no publication bias. Because the preparation of breath tests, collection of breath, storage of breath samples, and preprocessing sensor array data will influence the results of breath tests [16], current studies lack the standardization procedures of breath collection and machine learning analysis, which might cause heterogeneity from unknown sources. We suggested that a depository of analytical procedures before the implementation of statistical modeling might be essential to prevent heterogeneity in the diagnostic accuracy of breath tests.

### 4.3. Applicability of Findings to the Review Question

The current state of knowledge on the application of E-noses to clinical diagnosis remains uncertain. Although many types of sensors have been developed for many diseases, the accuracy of the E-nose is unclear because of the study design, patient selection, and lack of a standardized way to report the diagnostic accuracy. Ideally, a study should enroll a consecutive or random sample of eligible patients with the suspected disease to prevent the potential for bias [18]. However, in the clinical setting, when the prevalence of the disease is not high, researchers usually prefer to conduct a case-control study that enrolled participants with known disease and a control group without the condition that may increase the overall diagnostic accuracy [59]. If the disease prevalence of the research subjects included in the study is different from the target population, this will affect the applicability of the E-nose in the target population [18].

### 4.4. Limitations

Different studies used different machine learning algorithms, and the optimization procedure was not reported in most studies. The influence of accuracy might be affected by the type of sensor and statistical analyses. Furthermore, the limited sample size of the test sets may decrease the reported accuracy. Few studies had an independent test set to validate the test. This systematic review does not include studies with external validation tests; the pooled estimates of diagnostic accuracy from these studies cannot be generalized to other populations. We suggest that multicenter clinical studies among target populations with appropriate sample sizes and an independent validation set in different hospitals are crucial before an E-nose can be used in clinical applications.

### 4.5. Future Direction

There are several timings of breath tests in clinical practice. First, for patients who present with common nonspecific symptoms that could be an early indication of cancer, an exhaled breath test could act as a screening test. Second, for patients with suspected symptoms of diseases requiring further investigation, an E-nose can become a noninvasive point of care method before specialized investigations. Third, patients can receive therapy to detect disease recurrence. Current studies are focused on the first application. We suggest future studies for the second and third timings. Longitudinal studies are needed in the future to determine whether the electronic nose can be used to detect the recurrence of diseases.

The reproducibility of the results and reliability of instruments are future directions. Because E-nose studies are from diverse research fields, many researchers do not know how to provide essential items for reporting diagnostic accuracy studies. We recommend that future studies include clinical epidemiologists before implementing new breath tests to strengthen the study design, minimize the risk of bias and make the results more reliable. An E-nose is not capable of independently making a clinical diagnosis at this time. Physicians’ clinical diagnosis based on clinical symptoms, signs, laboratory tests, and pathological reports remain an essential requirement before starting therapy or surgery in the current stage.

A standard breath test must control the flow rate and humidity and collect alveolar air that contains the metabolites from the alveolar-capillary membrane and released into the alveolar space [52]. To improve the efficiency of the electronic nose breath test, we suggest that future research can continue to optimize the breath collection device, which can automatically control the flow rate and humidity and monitor the CO_2_ concentration to collect alveolar air containing volatile biomarkers.

## 5. Conclusions

Based on our meta-analysis, metal oxide sensors have good accuracy and may become important chip materials for electronic nose systems in the future. We encourage researchers currently using metal oxide sensors to conduct clinical trials to verify accuracy. In a breathomic study, case-control studies are suitable for exploring volatile biomarkers. However, in E-nose studies for diagnostic or screening purposes, studies that make inappropriate exclusions may result in an overestimation of diagnostic accuracy. The study ideally should enroll a consecutive or random sample of eligible patients with the suspected disease to prevent the potential for bias [18]. Machine learning techniques have gradually been applied in the medical field to establish a prediction model. However, many machine learning studies reported only the best accuracy value without showing details for readers to evaluate the reliability of test results. We suggest that studies should report the accuracy of the test set or an independent validation set. Researchers should not only show the best results with the highest accuracy; instead, a study should clearly explain all the procedures and conservatively estimate the accuracy for physicians in making clinical decisions [13].

## Figures and Tables

**Table 1:** The table displays for each included study.

Study	Disease	Sensor	Case	Control	Sensitivity	Specificity
Mommers [26], 2020	Aneurysm and recurrent hernia	Metal-oxide	64 ^a^	74	0.81	0.73
Wong [27], 2019	Appendicitis	Conductive polymer	5	45	0.8	0.8
Montuschi [28], 2010	Asthma	Quartz microbalances	30	21	0.87	0.95
Barash [29], 2015	Breast cancer	Gold nanoparticles and carbon nanotubes	169	82	0.88	0.83
Yang [5], 2021	Breast cancer	Carbon nanotubes	70 ^b^	18 ^a^	0.86 ^b^	0.97 ^b^
Fielding [30], 2020	Bronchial and laryngeal cancer	Carbon nanotubes	42	13	0.95	0.69
Amal [6], 2016	Colorectal cancer	Gold nanoparticles and carbon nanotubes	20 ^b^	36 ^b^	0.94 ^b^	0.91 ^b^
Shafiek [31], 2015	COPD	Carbon nanotubes	124	30	0.69	0.75
Binson [10], 2021	COPD and lung cancer	Metal-oxide	70	144	0.81	0.94
Welearegay [32], 2018	Cutaneous leishmaniasis	Metal nanoparticles	28 ^a^	28	0.96	1
Welearegay [33], 2019	Echinococcosis	Metal nanoparticles	36	40	0.97	0.98
van Dartel [34], 2020	Epilepsy	Metal-oxide	74	110	0.76	0.67
Broza [8], 2019	Gastric cancer	Gold nanoparticles	102	1065	0.82	0.79
Xu [35], 2013	Gastric cancer	Gold nanoparticles and carbon nanotubes	37	93	0.89	0.9
Leja [36], 2021	Gastric cancer	Gold nanoparticles	47	105	0.92	0.86
Umapathy [37], 2019	Haemodialysis	Metal-oxide	21	17	0.86	0.29
Gruber [38], 2014	Head and neck cancer	Nanomaterial-based sensor	22	19	0.77	0.9
Leunis [39], 2014	Head and neck cancer	Metal-oxide	36	23	0.9	0.8
Hakim [9], 2011	Head-and-neck cancer and lung cancer	Gold nanoparticles	36 ^a^	52	1	0.92
Finamore [40], 2018	Heart failure	Quartz microbalances	30 ^b^	39 ^b^	0.8 ^b^	0.82 ^b^
Moor [11], 2021	Interstitial lung disease	Metal-oxide	322	48	1	1
De Vincentis [12], 2016	Liver cirrhosis	Quartz microbalances	58	56	1	0.98
Zaim [41], 2021	Liver cirrhosis	WO3 nanowires	22	32	0.97	1
Gasparri [42], 2016	Lung cancer	Quartz microbalances	72	74	0.88	1
Huang [4], 2018	Lung cancer	Carbon nanotubes	56	188	0.92	0.93
Hubers [43], 2014	Lung cancer	Carbon nanotubes	38	39	0.87	0.43
Kononov [44], 2020	Lung cancer	Metal-oxide	19 ^b^	17 ^b^	0.95 ^b^	1 ^b^
Rocco [45], 2016	Lung cancer	Quartz microbalances	23	77	0.86	0.95
Shlomi [46], 2017	Lung cancer	Gold nanoparticles and carbon nanotubes	16	30	0.75	0.93
Tan [47], 2016	Lung cancer	Metal-oxide	12	13	0.83	0.88
Broza et al. [48], 2017	Multiple sclerosis	Gold nanoparticles	128	58	0.76	0.81
Nakhleh et al. [49], 2015	Parkinson’s disease	Gold nanoparticles and carbon nanotubes	16	37	0.81	0.76
Ionescu et al. [50], 2011	Multiple sclerosis	Polycyclic aromatic hydrocarbons and single-wall carbon nanotubes	34	17	0.85	0.71
Amal et al. [51], 2015	Ovarian cancer	Gold nanoparticles and carbon nanotubes	48	48	0.85	0.65
Raspagliesi et al. [7], 2020	Ovarian cancer	Metal-oxide	86	114	0.98	0.95
Yang et al. [52], 2018	Pneumoconiosis	Carbon nanotubes	34	64	0.68	0.84
Nakhleh et al. [53], 2016	Preeclampsia	Gold nanoparticles	31	31	0.92	0.91
Broza et al. [54], 2018	Rhinosinusitis	Gold nanoparticles and carbon nanotubes	17	30	0.76	0.8
Zamora-Mendoza et al. [55], 2022	SARS-CoV-2	Carbon nanotubes	42	30	0.97	1
Shan et al. [14], 2020	SARS-CoV-2	Gold nanoparticles	41	57	1	0.81
Wintjens et al. [56], 2020	SARS-CoV-2	Metal-oxide	57	162	0.86	0.54
Tsai et al. [57], 2021	Small airway dysfunction	Carbon nanotubes	12	60	0.92	0.95
Chen et al. [13], 2020	Ventilator-associated pneumonia	Carbon nanotubes	33	26	0.72	0.77
Schnabel et al. [58], 2015	Ventilator-associated pneumonia	Metal-oxide	33	53	0.88	0.66

^a^ Included data from the model for two disease outcomes. ^b^ Data derived from a test database.

**Table 2:** Subgroup analysis based on the type of sensor.

Type ^1^	Sensitivity (95% CI)	I^2^	Specificity (95% CI)	I^2^
Carbon nanotube (n = 8)	0.86 (0.75, 0.93)	69.4%	0.86 (0.71, 0.94)	82.1%
Conductive polymer (n = 1)	0.80 (0.31, 0.97)	NA	0.80 (0.66, 0.89)	NA
Gold nanoparticles (n = 6)	0.94 (0.80, 0.98)	39.8%	0.83 (0.78, 0.88)	48.5%
Gold nanoparticles and carbon nanotube (n = 6)	0.86 (0.82, 0.90)	0.0%	0.87 (0.82, 0.91)	32.5%
Metal-oxide (n = 10)	0.91 (0.81, 0.96)	35.2%	0.81 (0.63, 0.91)	89.5%
Metal nanoparticles (n = 2)	0.97 (0.88, 099)	0.0%	0.99 (0.90, 1.00)	0.0%
Nanomaterial-based (n = 1)	0.77 (0.56, 0.90)	NA	0.89 (0.66, 0.97)	NA
Polycyclic aromatic hydrocarbons and single wall carbon nanotubes (n = 1)	0.85 (0.69, 0.94)	NA	0.71 (0.46, 0.87)	NA
Quartz microbalances (n = 4)	0.93 (0.81, 0.97)	0.0%	0.98 (0.93, 0.99)	0.0%
WO3 nanowires (n = 1)	0.97 (0.80, 1.00)	NA	1.00 (0.00–1.00)	NA

^1^ The type of sensor is based on the classification provided in the literature.
